# A blueprint for health technology assessment capacity building: lessons learned from Malta

**DOI:** 10.1017/S0266462324000072

**Published:** 2024-02-29

**Authors:** Katharina Abraham, Ingelin Kvamme, Sylvana Magrin Sammut, Simone de Vries, Tanya Formosa, Rudy Dupree, Isaac Corro Ramos, Wim Goettsch, Margreet Franken

**Affiliations:** 1 Institute for Medical Technology Assessment, Erasmus University Rotterdam, Rotterdam, The Netherlands; 2 Institute for Medical Technology Assessment, Erasmus Centre for Health Economics Rotterdam (EsCHER), Erasmus School of Health Policy & Management, Erasmus University Rotterdam, Rotterdam, The Netherlands; 3 Department for Policy in Health, Ministry for Health, St. Luke’s Hospital, Pietà, Malta; 4 National Health Care Institute, Diemen, The Netherlands; 5 Division of Pharmacoepidemiology and Clinical Pharmacology, WHO Collaborating Centre for Pharmaceutical Policy and Regulation, Utrecht University, Utrecht, The Netherlands

**Keywords:** capacity building, health technology assessment, blueprint, low HTA capacity countries: EU HTA regulation, limited health economic expertise

## Abstract

**Objectives:**

The development and strengthening of health technology assessment (HTA) capacity on the individual and organizational level and the wider environment is relevant for cooperation on HTAs. Based on the Maltese case, we provide a blueprint for building HTA capacity.

**Methods:**

A set of activities were developed based on Pichler et al.’s framework and the starting HTA capacity in Malta. Individual level activities focused on strengthening epidemiological and health economic skills through online and in-person training. On the organizational level, a new HTA framework was developed which was subsequently utilized in a shadow assessment. Awareness campaign activities raised awareness and support in the wider environment where HTAs are conducted and utilized.

**Results:**

The time needed to build HTA capacity exceeded the planned two years accommodating the learning progress of the assessors. In addition to the planned trainings, webinars supplemented the online courses, allowing for more knowledge exchange. The advanced online course was extended over time to facilitate learning next to the assessors’ daily tasks. Training sessions were added to implement the new economic evaluation framework, which was utilized in a second shadow assessment. Awareness by decision-makers was achieved with reports, posters, and an article on the current and developing HTA capacity.

**Conclusions:**

It takes time and much (hands-on) training to build skills for conducting complex assessment such as HTAs. Facilitating exchange with knowledgeable parties is crucial for succeeding as well as the buy-in of local managers motivating staff. Decision-makers need to be on-boarded for the continued success of HTA capacity building.

## Introduction

Recently, the European Commission, Council, and Parliament have agreed on a new regulation on collaboration on health technology assessment (HTA) among member states, including voluntary cooperation on economic aspects in HTA (1). This EU HTA regulation (EU HTA-R) imposes a great need for capacity building in countries with limited use of HTA. HTA capacity building can be defined as the development and strengthening of individuals and organizations understanding, contributing to, and utilizing HTA for health policies and decision (2). Raising awareness and support for HTA is encouraged in the environment it is to operate in. While guidelines and roadmaps for the implementation of HTA capacity building are available, literature is lacking on the practical approach of the implementation of capacity building activities (3–5).

The available level of resources and local HTA capacities determines the extent to which national HTA bodies can institutionalize the HTA process. In countries with limited HTA resources, national bodies may utilize HTA evidence produced by other European jurisdictions or EUnetHTA, succeeded by the EU HTA-R in January 2025, to support their assessments efficiently (6). Such an approach is labeled as a light touch HTA approach (also including accepting HTA evidence from the pharmaceutical sector), while, on the other end of the scale, a heavy HTA approach favors in-house production of HTA reports (7).

While many countries include some level of HTA in their decision-making, research reveals that the extent of inclusion is often limited (8). A recent study revealed that Malta can benefit from improvements in HTA capacity. Identified barriers of the local HTA’s process included the lack of available local pricing data, strong interdependencies between subsequent processes, and limited health economic (HE) expertise (8). In this paper, we present a blueprint for the development and application of HTA capacity building based on the lessons learned from the capacity building exercise in Malta.

## Methods

The Maltese capacity building project started at the end of 2018 for a planned duration of two years. To understand the HTA capacity level in Malta, a needs assessment was first conducted, followed by a SWOT analysis (8). With a SWOT analysis, the strengths (S), weaknesses (W), opportunities (O), and threats (T) of a system towards a certain goal are identified to improve, utilize, and navigate the internal and external factors before implementing a large change, such as capacity building. Stakeholder interviews (N=33) were conducted to identify these factors to the Maltese reimbursement system objectives of public health, financial sustainability, and equitability. More information on the methods can be found in Abraham and Franken (8). SWOT factors that could be tackled by the HTA unit were considered in the capacity building activities.

The planned activities for building HTA capacity in Malta addressed all three levels described by Pichler et al. (2): individual, organizational, and environmental level, and focused on the assessment of pharmaceuticals. The focus on the individual level was skill training, especially on HE that would allow the HTA assessors to perform assessments with a newly drafted HTA framework. With the new framework, the use of HTAs in Malta should become more effective at the organizational level. Training on the fundamental HE concepts was planned online and in-person. The foundational training modules were to be released online at the start of the project. To ensure the fundamental concepts were understood, two local training sessions were planned shortly after with the HTA assessors and other department staff, including management. The advanced online training modules were targeted at HTA assessors with the aim to learn the skills and gain the knowledge necessary for the pharmaco-economic assessment of the new HTA framework. The advanced training was developed by senior health economists, customized based on the findings of the needs assessment and feedback on the fundamental training. It was planned to be released over the course of one year to allow for sufficient study time in parallel to the assessors’ normal tasks. The new framework and skillset of the HTA assessors were planned to be tested by shadowing an assessment with the learnings to be discussed during an in-person meeting at the Dutch National Health Care Institute (ZIN). Given their close collaboration at EUnetHTA, ZIN provided technical expertise, shared national best practices and experiences in the capacity building exercise. The final skill training activity was planned as in-person on-the-job training at ZIN. On the environmental level, the focus was on awareness campaign activities to draw local and international attention to challenges encountered, and efforts done to improve the Maltese system. Activities included the dissemination of the needs assessment among stakeholders, participation in local and international symposiums and conferences, as well as the publication of the capacity building activities.

## Results

The results include an overview of the HTA capacity level at the beginning of the project to better relate the capacity building elements which are outlined subsequently.

### The starting level

At the individual level, the lack of technical knowledge to critically assess methods for relative effectiveness assessments (REAs) and HE evaluations were considered the main limitation to HTA capacity. All HTA assessors had a strong pharmaceutical background but were not sufficiently familiar with basic epidemiological and HE concepts. On the organizational level, a lack of detailed HTA guidelines and procedures was found. The Maltese HTA framework consisted of an application form, general standard operating procedures, and a rudimentary assessment template. The HTA unit did not utilize a guideline on the type of evidence required for clinical and economic evaluation, nor a method defined for gathering evidence and/or quality appraising of the evidence. HTA assessors commonly utilized NICE dossiers for relative effectiveness, as access to other sources such as journals was limited. However, evidence in the NICE submissions not always overlapped with the comparator(s) relevant to Malta. In such circumstances, considerable weight was given to the clinicians’ opinions on the technology by the Maltese appraisal committee. For the pharmaco-economic assessment, the budget impact (BI) was calculated and cost-effectiveness conclusions from HTA dossiers of other English-speaking countries were reported. Assessors had difficulties interpreting the cost-effectiveness conclusions due to a lack of understanding and transferability issues regarding costs, clinical pathways, and willingness to pay for additional health. Consequently, HTA reports would provide little answer to the appraising committee on the additional benefit and value for money of the new technology. In addition, no guidelines existed on budget and cost-effectiveness thresholds. The committee appraising the financial sustainability also felt limited in providing recommendations on the budgetary spending for new technologies based on the BI calculations owing to limited local data such as patient numbers and prices. On the environmental level, the lack of a legal framework to request economic evidence from marketing authorization holders (MAHs) limited Malta’s HTA capacity as evidence had to be generated by the assessors. A complete description of the Maltese system, the needs assessment and SWOT analysis impacting the reimbursement processes, and thus HTA capacity, are provided in Abraham and Franken (8).

### Building HTA capacity

**Figure 1.**
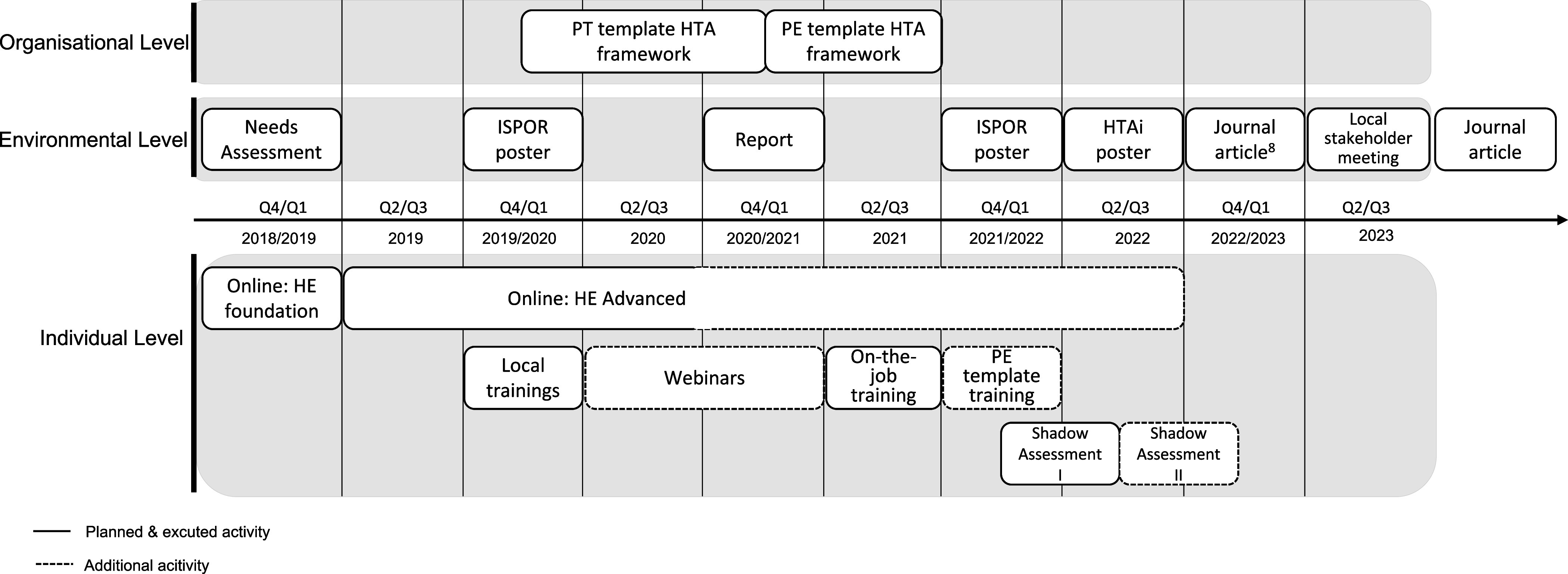
Planned and additional capacity building activities on the organizational, environmental, and individual level over the course of the project duration. HE, health economics; HTA, health technology assessment; PE, pharmaco-economic; PT, pharmaco-therapeutic.

The capacity building activities were mainly focused on the HTA unit and their Directorate (Figure 1). The HTA unit, consisting of four assessors including one team lead, falls under the Directorate for Pharmaceutical Affairs (DPA) within the Department Policy in Health led by the Chief Medical Officer. The technology assessments of the HTA unit are appraised by two committees within the Ministry for Health that provide reimbursement recommendations based on the added benefit and BI. The Minister for Health has the final responsibility for endorsing the decision of introducing a new medicine on the Government Formulary List.

#### Online HE training

The online HE training started as planned at the beginning of the project and consisted of 16 modules that staff could access at their own time and pace (i.e., asynchronous learning). The modules were developed by HTA experts who teach within a HE University Master program. Eight foundational modules covered the concepts on costs, cost-effectiveness, cost-effectiveness thresholds, quality-adjusted life years, and introduced biostatistics and epidemiology. In the eight advanced modules, the HTA assessors learned about regression analysis, strategies to control for confounding, evidence synthesis, quality of life (QoL) assessment, burden of illness, survival analysis, cost-effectiveness modeling, and uncertainty of cost-effectiveness results. The compulsory study materials included a total of 39 videos, 64 scientific articles, 13 readings, and 659 quiz questions. Passing all tests was needed for certification. The modules were released bi-monthly/quarterly, resulting in a spread of the online training over three years instead of one-and-a-half years to adjust for the onset of the pandemic and the assessors’ learning curves.

#### Local training

Two in-person training sessions took place in Malta at the end of 2019 and early 2020. The first training focused on the EUnetHTA framework to address the lack of HTA guidelines and procedures in Malta. EUnetHTA provides jointly produced HTA frameworks that can be utilized by countries for more alignment but also by countries with less experience in HTA. DPA participants were encouraged to derive learnings from the presented frameworks, including the HTA core model, population-intervention-comparator-outcomes approaches (PICO), and joint REAs and to deliberate their usefulness for the Maltese setting. In group exercises, the HTA assessors worked on deriving and discussing the PICO elements from a joint REA and their relevance for the Maltese setting. Based on feedback, prioritization approaches for HTAs to increase efficiency and impact of HTA (e.g., mini-HTA, cost-effectiveness analysis where added value is proven) were added to the session. The second local training aimed at solidifying the HE concepts learned in the online modules, such as identifying, measuring, and valuing costs and benefits. HTA assessors conducted multiple assignments on clinical effectiveness, QoL, costs, cost-effectiveness, and uncertainty of outcomes using the NICE single technology assessment. The local training revealed that the HTA assessors had no experience with Excel to facilitate economic evaluations and thus were advised to increase their skills.

In consultation with the local project managers, the initial number of skill training was extended to ensure key lessons were well understood. However, with the onset of the COVID pandemic, in-person training was no longer possible. Therefore, five additional webinars were organized synchronously to the online modules on QoL, survival analysis, HE modeling, and on uncertainty of outcomes. The webinars showed to be crucial to the development of the assessors’ skills as they could verify and deepen their learnings with health economists.

#### On-the-job training

The on-the-job training was held online due to ongoing travel restrictions. During the 4-day training, the Maltese assessors learned about the day-to-day work of the pharmaco-therapeutic and/-economic assessors at ZIN and performed an REA on a selected submission file for a health technology (i.e., dossier). In groups, the assessors critically assessed (a) the PICO and its application to the Maltese situation, (b) the selection of studies forming the evidence for the clinical effectiveness provided by the MAH, and (c) the treatment pathway of the technology. The Maltese assessors also participated as observers in a meeting with ZIN assessors discussing the challenges on HTA reports and procedures and attended a meeting at ZIN on setting priorities for evaluating pharmaceuticals.

#### Development of a new HTA framework

Begin 2020, the Maltese HTA assessors started developing the pharmaco-therapeutic assessment template for the new HTA framework based on the EUnetHTA framework. In early 2021, a draft version was shared for alignment with the pharmaco-economic assessment template developed by the Dutch institute of Medical Technology Assessment (iMTA). The new templates aimed at addressing the lack of a standard report structure, the need to discuss uncertainties, and recommendations on relative effectiveness. With the development of the pharmaco-economic assessment template, several fundamental choices had to be made. In Malta, both medical consultants and MAHs can submit applications. Consequently, the level of provided evidence could range from no evidence to evidence from global dossiers. Additionally, no procedures were in place to decide on a REA or a full HTA that includes an economic evaluation. Consequently, it was difficult to develop a pharmaco-economic assessment framework. Moreover, common HE elements specific to a country, such as the evaluation perspective and rate of discounting, had to be decided on but there were no local experts. Consequently, in consultation with ZIN and iMTA, the Maltese reference case for the economic analysis was built from the NICE reference case. DPA was further advised to consider a HTA framework similar to the Netherlands, namely, to only conduct full HTAs after an added benefit was concluded or a high BI was expected. Before the piloting of the new HTA framework, the assessors practiced with the pharmaco-economic template on a NICE dossier during five two-and-a-half hour-webinars.

#### Shadow assessment I

The dossier selected for the first shadow assessment was primarily based on the preference of DPA and secondarily, for efficiency reasons, on the familiarity with the technology to be assessed by the supporting agencies (iMTA, ZIN). The collaborating MAH provided a global BI model with data from the United Kingdom (UK) and clinical effectiveness literature. In addition, the HTA assessors utilized the NICE dossier and comments of the independent evidence review group that critically appraises the evidence submission to NICE. The shadow assessment was initiated end of 2021 during a 2-day online session. Guided by the newly developed template, the assessors conducted the REA for the new technology over a period of three months. During six online meetings, the HTA assessors presented their assessment and were asked questions on the technical aspects of their work. One main issue concerned the comparator and method of relative effectiveness. In the NICE dossier, a comparator not applicable to the Maltese setting was assessed with a matching-adjusted indirect comparison whereas the appropriate comparator was assessed in the ZIN dossier but with a naïve comparison. After information on the advantages and limitations of the two relative effectiveness methods were shared, the Maltese assessors concluded equal benefit of the new technology which resulted in a BI analysis only based on the new HTA framework. Although the MAH had provided a programmable BI model in Excel, the HTA assessors felt more confident to conduct the analysis manually. An additional online training was conducted working with the programmable BI model to strengthen the assessors’ confidence on more efficient ways of calculating but also to create awareness on the impact parameters can have. The final presentation on the REA included a role-play to test the new framework further by supporting HTA assessors when presenting to the appraising committee.

#### Shadow assessment II

Since the pharmaco-economic framework could not be tested in the first shadow assessment, a second shadow assessment was conducted focusing mainly on the pharmaco-economic part. Upon request, the collaborating MAH provided a global HE model. Over the course of several online meetings in mid-2022, the pharmaco-economic template was filled in and discussed in detail. The Maltese assessors found the pharmaco-economic section particularly challenging to understand and to complete despite receiving online training prior to the assessment indicating the steep learning curve associated with HE. The three initially planned online meetings were supplemented with several in-between online meetings including one to two HE experts. This allowed for informal discussions and assistance on the assessment. To finalize the result and discussion section of the pharmaco-economic assessment, a 3-day in-person training was organized in Malta. During this training, the assessors also gained experience in altering parameter inputs in the electronic model and building a hypothetical Maltese base-case, including country-specific costing data, gaining understanding in how changes in input parameters impact outcomes, and the uncertainties of outcomes. Each HTA assessor prepared and presented their conclusions on the pharmaco-economic evidence. The assessors experienced the second shadow assessment as complicated and expressed the need for adjustment considering the national situation, resources, and limited expertise.

#### Awareness campaign

Planned activities for the awareness campaign were initiated following the needs assessment report that caused the most impact on the environmental level. The report included a detailed description of the core processes of the Maltese reimbursement system, an analysis of the SWOT, and recommendations for the Ministry for Health. The report was shared with the local project leads towards the end of 2020 and was forwarded to the higher officials of the Ministry for Health where it generated awareness. Internal meetings with key stakeholders within the Ministry were initiated, and plans to continue prioritize the necessary actions were explored. Internationally, awareness was created with an article on the Maltese needs assessment, and at ISPOR 2019, ISPOR 2021, and HTAi 2022 with four poster presentations (8–12). Although participation in local symposiums was limited due to the pandemic, relevant local stakeholders convened in Malta in May 2023.

## Discussion

HTA capacity building activities should address the individual level (i.e., skill training on ability to perform HTA), organizational level (i.e., development of a new HTA framework to make the use of HTA’s more effective and efficient), and the environmental level (i.e., awareness campaign).

The Maltese capacity building exercise showed that the greatest gain was on the individual level. Thus, the capacity building activities focused on skills such as learning HE and epidemiological concepts, applying, and presenting the learned knowledge. While the study by Rabayah et al. (3) found limited skills in Excel and report writing, time constraints, and staff retention to be the main limitations for HTA capacity building; in the Maltese setting, it was subject knowledge. Staff was retained throughout the duration of the project despite the steep learning curve and obstacles on the organizational and environmental levels (e.g., lack of data). The assessors displayed a high level of motivataction to gain further knowledge on relative effectiveness methods and to improve their HE skills. This could be contributed to the local project leads who continued to motivate and encourage their staff throughout the five years of the project. Thus, countries who are building their HTA skills and experience difficulties with staff retention may benefit from a motivated local project lead. Due to the onset of the COVID pandemic, timelines on capacity building were postponed extending the project far beyond the planned two years. However, the longer time horizon benefited the capacity building process as the true HTA capacity was revealed only over time due to the broad scope of skills needed. Countries with initial low HTA skills, likely benefit from a longer capacity building time horizon to ensure fundamental HE concepts are well understood while their daily activities are continued. While the Maltese assessors also displayed limited Excel skill, contrary to what was found by Rabayah et al., it did not significantly limit capacity building. Although a fundamental Excel course was advised for utilizing more advanced calculation tools, capacity building should not be postponed, therefore. During the shadow assessment exercises, the Maltese assessors were trained on the new HTA framework in close communication with HTA experts and assessors from the Netherlands. Gaining knowledge on the uncertainty of modeling relative (cost-) effectiveness, understanding the potential causes of the uncertainty, and exchanging on the day-to-day work with other assessors were found to be crucial to the assessors’ confidence in conducting their own assessments and in the communication of HTA outcomes. Therefore, countries who aim at building their HTA capacity are advised to include close exchanges with HTA assessors from other jurisdictions with well-established HTA capacity in the skills training.

On the organizational level, the drafting of a local HTA framework aimed at facilitating the use of HTA evidence in the local setting by tackling transferability issues regarding relative (cost-) effectiveness and by standardizing reports to improve quality. For the pharmaco-therapeutic assessment, countries who struggle with standardization can utilize EUnetHTA’s template for rapid REAs. While the Core Model can be implemented directly by countries (although potentially too extensive for countries with very limited resources), standard pharmaco-economic templates and HE guidelines are still lacking as they are country-specific. Since the choices for a pharmaco-economic framework are dependent on national preferences and cannot be implemented bottom up, countries with limited resources are advised to base their reference case for the pharmaco-economic assessment on jurisdictions similar to them. To improve efficiency and account for limited staff availability and time constraints, countries could opt to only request full HTAs based on BI and/or added therapeutic benefit, similar to the Netherlands. For producing HTA evidence, a heavy touch in-house production was not considered feasible for low resource countries. However, the direct reuse of HTA evidence from other jurisdictions (light touch approach), especially with regard to the pharmaco-economic part may result in transferability issues (6). For clinical effectiveness, EUnetHTA’s joint reports pose an opportunity for a successful light touch approach. From 2025 onward, the HTA-R will become mandatory for European countries starting with oncology and advanced therapy medicinal products (1). This regulation promotes the inclusion of the relevant populations and comparators of EU countries in the centrally conducted clinical effectiveness assessments which especially supports countries with limited resources. Assessments outside the mandatory and voluntary cooperation, but especially for cost-effectiveness assessments, HTA reports from countries with similar healthcare system structure could be utilized, like NICE reports for Malta. A resource-efficient approach for countries with limited staff availability, time constraints, or limited interest from MAHs could be to critically review (economic) parameters and input data regarding variations between a reference country and local setting, such as the UK for Malta. In addition, relevant uncertainties on relative (cost-) effectiveness outcomes from the reported sensitivity analyses could be discussed. Nevertheless, locally adapted economic evaluations should be required from MAHs for currency, inflation, and adjustment of costing parameters to the local setting. The capacity building exercise showed that local costing parameters can be derived, in the case of Malta e.g., from the main local hospital. Even if not yet validated, it is taking the first step towards a more country-specific HTA. Countries with constraints on costing data may take similar steps to facilitate a country-specific approach. From the capacity building in Malta, we learned that through facilitated HTA training, the assessors became better equipped to appropriately scrutinize and provide relevant evidence to the decision-makers and therefore improved their local HTA processes.

While cost-effectiveness evidence was an official criterion in Malta as well as in many other European countries who are building HTA capacity, its implementation is limited when the legal frameworks do not impose it as a mandatory requirement. Agents who can influence or impose change to the legal frameworks, therefore, are crucial, so efforts conducted on the individual and organizational level are further facilitated, and HTA capacity is further improved. Although awareness at the environmental level can be created with scientific reports and presentations, decision-makers need to implement the requirements for a successful HTA process (e.g., mandatory HTA evidence, cost-effectiveness and budget thresholds, assessment perspective, discounting, etc.). Furthermore, continued skills attainment on the individual level is vital including training of potential new HTA staff and all committees who utilize HTA evidence. While this blueprint for HTA capacity building focused on the assessment of pharmaceuticals, there is a whole range of other health technologies, such as medical devices and in vitro diagnostic, that likely require additional evaluation skills and frameworks. Other learnings from this study were to keep timelines flexible, given unexpected external events (e.g., a pandemic) and staff time restriction, the time needed to reveal the true capacity level and therefore, adjustments in the capacity building activities. Furthermore, it was noticed that investments in communication skills may also be beneficial to improve communication for the purpose and outcomes of HTA to both internal and external stakeholders.

## Conclusion

Capacity building activities that can be implemented bottom-up have great potential but need continued support from management to keep staff motivated and focused as change is challenging and takes time. A sustained effort on building HTA capacity on the individual and organizational level, and by creating awareness on the environmental level together with strong leadership supporting HTA, as shown in Malta, achieving HTA capacity is feasible and achievable.

## Data Availability

Data sharing not applicable to this article as no datasets were generated or analyzed during the current study.
